# Vasopressin improves survival in a porcine model of abdominal vascular injury

**DOI:** 10.1186/cc5977

**Published:** 2007-07-23

**Authors:** Karl H Stadlbauer, Horst G Wagner-Berger, Anette C Krismer, Wolfgang G Voelckel, Alfred Konigsrainer, Karl H Lindner, Volker Wenzel

**Affiliations:** 1 Department of Anaesthesiology and Critical Care Medicine, Innsbruck Medical University, Anichstrasse, 6020 Innsbruck, Austria; 2 Department of Surgery, Eberhard-Karls Unversity, Hoppe-Seyler-Straße, 72076 Tübingen, Germany

## Abstract

**Introduction:**

We sought to determine and compare the effects of vasopressin, fluid resuscitation and saline placebo on haemodynamic variables and short-term survival in an abdominal vascular injury model with uncontrolled haemorrhagic shock in pigs.

**Methods:**

During general anaesthesia, a midline laparotomy was performed on 19 domestic pigs, followed by an incision (width about 5 cm and depth 0.5 cm) across the mesenterial shaft. When mean arterial blood pressure was below 20 mmHg, and heart rate had declined progressively, experimental therapy was initiated. At that point, animals were randomly assigned to receive vasopressin (0.4 U/kg; *n *= 7), fluid resuscitation (25 ml/kg lactated Ringer's and 25 ml/kg 3% gelatine solution; *n *= 7), or a single injection of saline placebo (*n *= 5). Vasopressin-treated animals were then given a continuous infusion of 0.08 U/kg per min vasopressin, whereas the remaining two groups received saline placebo at an equal rate of infusion. After 30 min of experimental therapy bleeding was controlled by surgical intervention, and further fluid resuscitation was performed. Thereafter, the animals were observed for an additional hour.

**Results:**

After 68 ± 19 min (mean ± standard deviation) of uncontrolled bleeding, experimental therapy was initiated; at that time total blood loss and mean arterial blood pressure were similar between groups (not significant). Mean arterial blood pressure increased in both vasopressin-treated and fluid-resuscitated animals from about 15 mmHg to about 55 mmHg within 5 min, but afterward it decreased more rapidly in the fluid resuscitation group; mean arterial blood pressure in the placebo group never increased. Seven out of seven vasopressin-treated animals survived, whereas six out of seven fluid-resuscitated and five out of five placebo pigs died before surgical intervention was initiated (*P *< 0.0001).

**Conclusion:**

Vasopressin, but not fluid resuscitation or saline placebo, ensured short-term survival in this vascular injury model with uncontrolled haemorrhagic shock in sedated pigs.

## Introduction

For haemodynamic stabilization of critically injured patients with uncontrolled haemorrhagic shock, current advanced trauma life support guidelines recommend infusion of crystalloid or colloid solutions. Interestingly, there appears to be no evidence either for or against early or large amounts of intravenous fluid administration in uncontrolled haemorrhage [1,2]. Although these findings on fluids in uncontrolled haemorrhagic shock are inconclusive, the strategy of delaying fluid resuscitation must be deemed unconfirmed as well, because the only study to examine the efficacy of this approach yielded barely significant findings.

One way to promote vasoconstriction may be to inject vasopressin, which has potent vasoconstrictive effects, even in severe acidosis and developed vasoplegia. In porcine models of uncontrolled haemorrhagic shock after liver trauma, vasopressin was superior to fluid resuscitation, adrenaline (epinephrine), and saline placebo in terms of blood loss, haemodynamic variables, and survival [3-5]. In agreement with these experiments, vasopressin reduced blood loss and stabilized arterial blood pressure in patients who had suffered traumatic or nontraumatic injury with haemorrhagic shock that was refractory to catecholamines [6-9]. Although beneficial effects of vasopressin may seem realistic in a parenchymatic organ such as the liver, pronounced vascular injury may impose limitations with regard to vasoconstriction-mediated reductions in blood loss. This may be important because patients suffering multiple trauma often sustain complex injuries, with bleeding from multiple sources. A clinical trial comparing vasopressin against placebo as an adjunct to standard shock treatment in unstable multiple trauma patients is currently in preparation [10], but further information about underlying mechanisms is needed.

The purpose of the present study was to compare the effects of vasopressin with those of fluid resuscitation and saline placebo on haemodynamic variables and short-term survival in an abdominal vascular injury model of uncontrolled haemorrhagic shock. Our null hypothesis was that there would be no differences in study end-points.

## Materials and methods

### Surgical preparations and measurements

The project was approved by the Austrian Federal Animal Investigational Committee, and the animals were managed in accordance with the American Physiological Society institutional guidelines and the Position of the American Heart Association on Research Animal Use, as adopted on 11 November 1984. Animal care and use were performed by qualified individuals and under the supervision of veterinarians, and all facilities and transportation complied with current legal requirements and guidelines. Anaesthesia was used in all surgical interventions, any unnecessary suffering was avoided and research was terminated if unnecessary pain or stress resulted. Our animal facilities meet the standards of the American Association for Accreditation of Laboratory Animal Care.

This study was conducted in 19 healthy swine, aged 12 to 16 weeks and weighing 30 to 40 kg. The animals were fasted overnight, but they had free access to water. The pigs were pre-medicated with azaperone (4 mg/kg intramuscularly) and atropine (0.1 mg/kg intramuscularly) 1 hour before surgery, and anaesthesia was induced with propofol (1 to 2 mg/kg intravenously). After intubation during spontaneous respiration, the pigs were ventilated using a volume controlled ventilator (Draeger EV-A, Lübeck, Germany) with 35% oxygen at 20 breaths/minute, 5 cmH_2_O positive end-expiratory pressure, and tidal volume adjusted to maintain normocapnia. Anaesthesia was maintained with propofol (6 to 8 mg/kg per hour) and a single injection of piritramide (30 mg) [11]. Muscle paralysis was achieved with 0.2 mg/kg per hour pancuronium after intubation, in order to facilitate laparotomy. Lactated Ringer's solution (250 ml) and a 3% gelatine solution (250 ml; Gelofusin^®^, B. Braun, Melsungen, Germany) were administered during the preparation phase. A standard lead II electrocardiograph was used to monitor cardiac rhythm; depth of anaesthesia was judged according to blood pressure, heart rate and electroencephalography (Neurotrac; Engström, Munich, Germany).

If cardiovascular variables or electroencephalography indicated reduced depth of anaesthesia, then additional propofol and piritramide were given. Body temperature was maintained between 38.0°C (100.4°F) and 39.0°C (102.2°F). A 7-Fr catheter was advanced into the descending aorta via a femoral cut-down for withdrawal of arterial blood samples and measurement of arterial blood pressure. A 7.5-Fr pulmonary artery catheter was placed via cut-down in the neck for measurement of right atrial and pulmonary artery pressures. Blood pressure was measured using a saline-filled catheter attached to a pressure transducer (model 1290A; Hewlett Packard, Böblingen, Germany), which was calibrated to atmospheric pressure at the level of the right atrium. Pressure tracings were recorded using a data acquisition system (Dewetron port 2000 [Dewetron, Graz, Austria] and Datalogger [custom software]). Blood gases were measured using a blood gas analyzer (Rapidlab 865; Chiron, Walpole, MA, USA); end-tidal carbon dioxide was measured using an infrared absorption analyzer (Multicap, Datex, Helsinki, Finland).

### Experimental protocol

**Figure 1:**
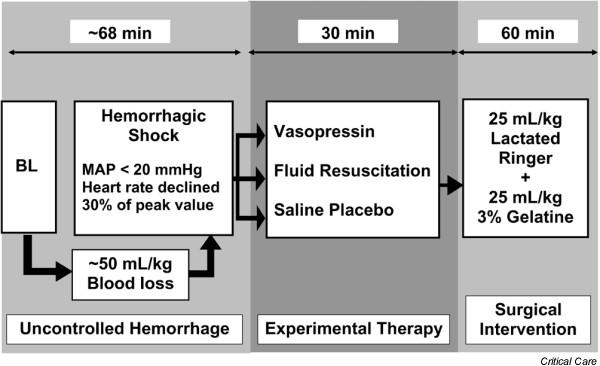
Flow chart of the experimental protocol. BL, baseline; MAP, mean arterial pressure.

Figure 1 provides a summary of the experimental protocol. After assessing baseline haemodynamic values, a midline laparotomy was performed. Propofol infusion was adjusted to 2 mg/kg per hour, and infusion of lactated Ringer's and gelatine solution was stopped before initiation of the experiment. During uncontrolled haemorrhage and experimental therapy, tidal volume was not adjusted. An incision (width 5 cm and depth 0.5 cm) was made across the mesenterial shaft. When mean arterial blood pressure was below 20 mmHg and heart rate had declined by more than 30% of its peak value, pharmacological support was provided for 30 min to simulate a pre-hospital phase before surgical intervention.

At that point, the 19 animals were randomly assigned to receive one of the following: 0.4 U/kg vasopressin (Pitressin^®^; Parke-Davis/Pfizer, Karlsruhe, Germany) diluted to 20 ml with saline (*n *= 7); fluid resuscitation (25 ml/kg lactated Ringer's and 25 ml/kg 3% gelatine solution; *n *= 7); or a single injection of 20 ml saline placebo (*n *= 5). Fluid resuscitation was initially set at about 2 ml/kg per min over the first 10 min. If this approach failed to restore arterial blood pressure, then fluid resuscitation was enhanced to about 8 ml/kg per min. We used a combination of Ringer's lactate and gelatine solution for fluid resuscitation, because it is the usual strategy in Europe. Vasopressin-treated animals were then given a continuous infusion of 0.08 U/kg per min vasopressin, whereas the remaining two groups received saline placebo at an equal rate of infusion.

After initiating experimental therapy, pigs were ventilated with 100% oxygen. The investigators were blinded as to the treatment given to each pig. To achieve blinding, we employed three-way stopcocks, which directed experimental treatment either into a covered bucket or into the central venous line. The infusion rate of fluids was set in all groups to 2 ml/kg at the beginning of the experimental therapy. A three-way stopcock determined whether subsequent experimental therapy was directed into the central venous line (fluid-resuscitated animals) or into a covered bucket (vasopressin-treated and placebo-treated animals). Hence, our vasopressin-treated and placebo-treated animals received no additional fluid during experimental therapy. The infusion rate was enhanced to 8 ml/kg per min after 10 min of experimental therapy if mean arterial pressure did not increase to above 40 mmHg. When mean arterial blood pressure reached aortic hydrostatic pressure (about 10 mmHg) and end-tidal carbon dioxide was 10 mmHg or less, the animals were declared dead and administered an overdose of fentanyl, propofol and potassium chloride.

After 30 min of experimental therapy, bleeding was controlled by surgical intervention in all surviving pigs. Additionally, fluid therapy (25 ml/kg lactated Ringer's and 25 ml/kg 3% gelatine solution) was started, and haemodynamic variables and blood gases were measured over an additional observation period of 60 min. Afterward, the pigs were killed as described above.

### Statistical analysis

Values are expressed as mean ± standard deviation. The comparability of baseline data was verified using one-way analysis of variance. Survival rates were compared using Kaplan-Meier methods with log rank (Mantel Cox) comparison of cumulative survival by treatment group, and were corrected using the Bonferroni method for multiple comparisons. Differences with a two-tailed *P *value < 0.05 were considered significant. Because of rapidly changing number of surviving animals in the experimental therapy phase, we did not perform further statistical analysis.

## Results

**Figure 2:**
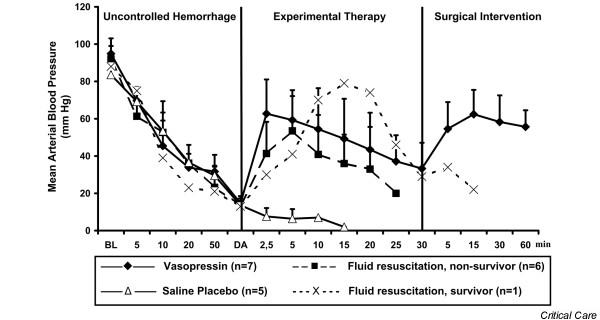
Mean arterial blood pressure. Values are expressed as mean (± standard deviation) arterial blood pressure before, during and after administration of a 0.4 U/kg bolus dose and 0.08 U/kg per min continuous infusion of vasopressin (*n *= 7), fluid resuscitation (divided into survivors [*n *= 1] and nonsurvivors [*n *= 6]), and saline placebo (*n *= 5). 'Uncontrolled haemorrhage' indicates the non-intervention interval after vessel injury; 'experimental therapy' indicates vasopressin treatment, fluid resuscitation, or saline placebo administration without bleeding control; and 'surgical intervention' indicates surgical management of the mesenteric shaft to control bleeding. The x-axis does not reveal the true time slope. BL, baseline; DA, drug administration.

**Figure 3:**
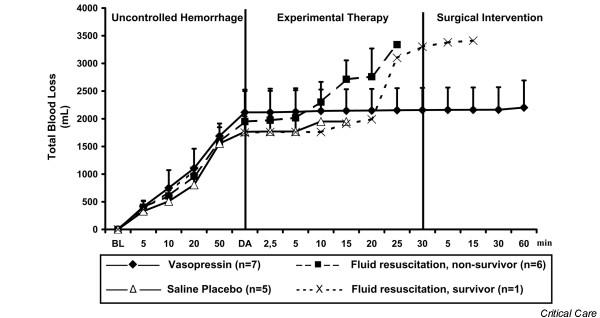
Total blood loss. Values are expressed as mean (± standard deviation) total blood loss before, during, and after administration of a 0.4 U/kg bolus dose and 0.08 U/kg per min continuous infusion of vasopressin (*n *= 7), fluid resuscitation (divided into survivors [*n *= 1] and nonsurvivors [*n *= 6]), and saline placebo (*n *= 5). 'Uncontrolled haemorrhage' indicates the non-intervention interval after vessel injury; 'experimental therapy' indicates vasopressin treatment, fluid resuscitation, or saline placebo administration without bleeding control; and 'surgical intervention' indicates surgical management of the mesenteric shaft to control bleeding. The x-axis does not reveal the true time slope. BL, baseline; DA, drug administration.

Before induction of haemorrhage, there were no differences in haemodynamic variables, blood gases, weight, or temperature between groups. Experimental therapy was initiated after 76 ± 24 min in the vasopressin group, 63 ± 19 min in the fluid resuscitation group and 64 ± 9 min in the saline placebo group (not significant). At that time, mean arterial blood pressure (Figure 2) and total blood loss (Figure 3) were comparable between groups. Before drug administration, lactate and arterial carbon dioxide tension were significantly lower in the saline placebo group than in the vasopressin group (Table 1).

**Table 1:** Arterial blood gas variables, haemoglobin, and lactate

Parameter	Group	Baseline	Haemorrhagic shock	Experimental therapy		Surgical intervention	
				
				5 min after DA	30 min after DA	15 min	60 min
				7.34 ± 0.12	7.18 ± 0.10	7.08 ± 0.12	7.21 ± 0.13
	Fluid resuscitation	7.50 ± 0.05	7.36 ± 0.08	7.17 ± 0.03	-	-	-
	Saline placebo	7.52 ± 0.01	7.44 ± 0.08	7.41 ± 0.06	-	-	-
Paco_2 _(mmHg)	Vasopressin	37 ± 3	31 ± 4	30 ± 3	34 ± 5	44 ± 4	41 ± 5
	Fluid resuscitation	37 ± 3	30 ± 5	45 ± 7	-	-	-
	Saline placebo	34 ± 3	24 ± 4*	24 ± 7	-	-	-
Pao_2 _(mmHg)	Vasopressin	154 ± 19	132 ± 21	312 ± 134	437 ± 31	363 ± 102	262 ± 98
	Fluid resuscitation	138 ± 20	123 ± 28	300 ± 168	-	-	-
	Saline placebo	154 ± 10	116 ± 25	239 ± 131	-	-	-
Base excess (mmol/l)	Vasopressin	5.7 ± 1.7	-9.0 ± 4.5	-8.8 ± 5.8	-14.1 ± 6.1	-15.2 ± 5.0	-10.5 ± 6.0
	Fluid resuscitation	5.3 ± 3.8	-7.7 ± 3.1	-11.4 ± 1.4	-	-	-
	Saline placebo	4.5 ± 1.8	-7.5 ± 3.4	-8.9 ± 3.3	-	-	-
Haemoglobin (g/dl)	Vasopressin	9.0 ± 1.2	7.7 ± 0.9	7.2 ± 1.0	6.4 ± 1.3	4.2 ± 0.7	3.3 ± 1.1
	Fluid resuscitation	8.5 ± 0.9	7.2 ± 1.5	4.4 ± 1.4	-	-	-
	Saline placebo	8.4 ± 0.9	8.1 ± 0.5	7.8 ± 0.7	-	-	-
Lactate (mmol/l)	Vasopressin	1.78 ± 0.33	9.88 ± 2.78	11.10 ± 3.11	13.10 ± 3.44	11.55 ± 3.89	9.88 ± 3.55
	Fluid resuscitation	2.00 ± 1.22	7.55 ± 2.55	7.33 ± 2.66	-	-	-
	Saline placebo	1.22 ± 0.22	5.99 ± 1.44*	8.44 ± 2.66	-	-	-

All variables are expressed as mean ± standard deviation. 'Baseline' indicates measurements taken before mesenteric vessel trauma; 'haemorrhagic shock' indicates values taken at the point of experimental intervention (mean arterial pressure <20 mmHg and heart rate <30% of its peak value); and 'experimental therapy' indicates vasopressin, fluid resuscitation, or saline placebo administration without bleeding control. Statistical comparison was only performed at baseline and haemorrhagic shock: *P < 0.05 for placebo versus vasopressin. -, not applicable because of death in fluid-resuscitated and placebo-treated animals; DA, drug administration; Paco_2_, arterial carbon dioxide tension; Pao_2_, arterial oxygen tension.

**Figure 4:**
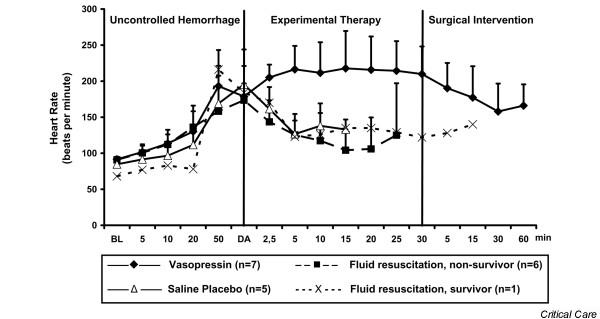
Heart rate. Values are expressed as mean (± standard deviation) heart rate before, during, and after administration of a 0.4 U/kg bolus dose and 0.08 U/kg per min continuous infusion of vasopressin (*n *= 7), fluid resuscitation (divided into survivors [*n *= 1] and nonsurvivors [*n *= 6]), and saline placebo (*n *= 5). 'Uncontrolled haemorrhage' indicates the non-intervention interval after vessel injury; 'experimental therapy' indicates vasopressin treatment, fluid resuscitation, or saline placebo administration without bleeding control; and 'surgical intervention' indicates surgical management of the mesenteric shaft to control bleeding. The x-axis does not reveal the true time slope. BL, baseline; DA, drug administration.

**Figure 5:**
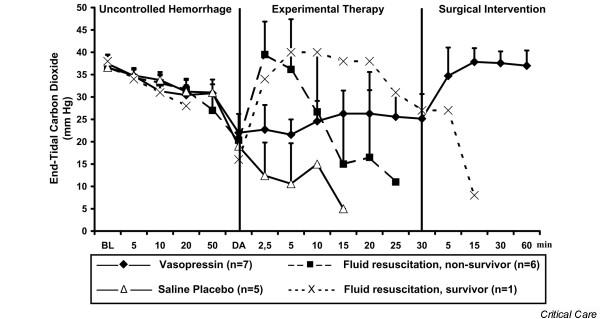
End-tidal carbon dioxide. Values are expressed as mean (± standard deviation) end-tidal carbon dioxide before, during, and after administration of a 0.4 U/kg bolus dose and 0.08 U/kg per min continuous infusion of vasopressin (*n *= 7), fluid resuscitation (divided into survivors [*n *= 1] and nonsurvivors [*n *= 6]), and saline placebo (*n *= 5). 'Uncontrolled haemorrhage' indicates the non-intervention interval after vessel injury; 'experimental therapy' indicates vasopressin treatment, fluid resuscitation, or saline placebo administration without bleeding control; and 'surgical intervention' indicates surgical management of the mesenteric shaft to control bleeding. The x-axis does not reveal the true time slope. BL, baseline; DA, drug administration.

**Figure 6:**
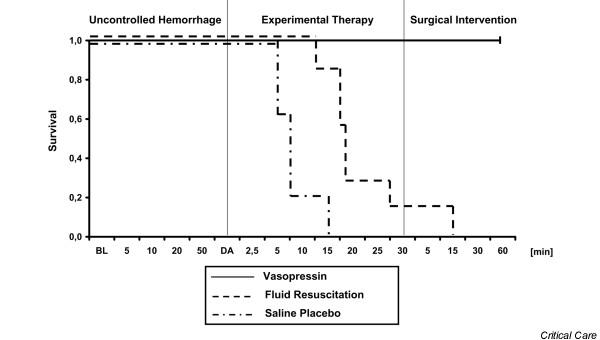
Kaplan-Meier survival curves. Shown are Kaplan-Meier survival curves before, during, and after administration of a 0.4 U/kg bolus dose and 0.08 U/kg per min continuous infusion of vasopressin (*n *= 7), fluid resuscitation (*n *= 7), and saline placebo (*n *= 5). 'Uncontrolled haemorrhage' indicates the non-intervention interval after vessel injury; 'experimental therapy' indicates vasopressin treatment, fluid resuscitation, or saline placebo administration without bleeding control; and 'surgical intervention' indicates surgical management of the mesenteric shaft to control bleeding. The x-axis does not reveal the true time slope. *P *< 0.0001. BL, baseline; DA, drug administration.

After initiating experimental therapy, the heart rate remained at about 210 beats/min in the vasopressin group, but it decreased from about 180 to about 120 beats/min in the fluid resuscitation and saline placebo groups (Figure 4). Mean arterial blood pressure increased in both vasopressin-treated and fluid-resuscitated animals from about 15 mmHg to about 55 mmHg within 5 min of experimental therapy, but it decreased immediately in placebo-treated animals. Mean arterial blood pressure declined more rapidly in the fluid resuscitation group than in vasopressin-treated swine after 5 min of experimental therapy (Figure 2). End-tidal carbon dioxide remained at about 25 mmHg in the vasopressin-treated group, but it decreased rapidly in the placebo group. In fluid-resuscitated animals, end-tidal carbon dioxide increased from about 20 mmHg to 40 mmHg within 5 min, but it subsequently deteriorated (Figure 5). Total blood loss was constant in the vasopressin and saline placebo groups, but it increased in the fluid resuscitation group from about 2,000 ml to about 2,800 ml (Figure 3). Within the first 5 min of experimental therapy, haemoglobin was constant in the vasopressin and saline placebo groups, but it decreased in the fluid resuscitation group from about 7.2 to 4.4 g/dl (Table 1). Seven out of seven vasopressin-treated animals survived, whereas six out of seven fluid-resuscitated and five out of five placebo-treated pigs died before surgical intervention was initiated (Figure 6; *P *< 0.0001).

## Discussion

In this porcine model of vascular injury of uncontrolled haemorrhagic shock, vasopressin maintained cardiocirculatory function at a level that was sufficient to permit at least short-term survival. In contrast, within 20 min of experimental therapy, six out of seven fluid-resuscitated and five out of five placebo-treated animals died.

Bleeding was initiated in this model of uncontrolled haemorrhagic shock by an incision to the mesenteric shaft. Thus, we simulated a blood vessel injury, which is often associated with blunt trauma and a subsequent high mortality rate [12]. Dosages employed in the experimental strategies were similar to those in interventions in an established porcine liver trauma model involving uncontrolled haemorrhagic shock [4,5]. Accordingly, we suggest that this model of uncontrolled haemorrhagic shock, caused by a blood vessel injury, is a valuable tool in which to assess the effect of experimental advanced trauma life support on haemodynamic variables and short-term survival.

Vasopressin has been investigated in various catecholamine-refractory shock states, such as cardiac arrest [13-15] and vasodilatory shock [16-18]. For example, even in normovolaemic but catecholamine-refractory haemorrhagic shock, vasopressin effectively restored cardiocirculatory function in a canine model [19]. That report is in full agreement with the findings of our study, in which vasopressin rapidly increased mean arterial blood pressure from about 15 mmHg to about 50 mmHg after approximately 65 min of uncontrolled bleeding (total blood loss about 50 ml/kg), resulting in severe haemorrhagic shock. This vasopressin-mediated increase in mean arterial blood pressure was also observed in trauma patients with uncontrolled haemorrhagic shock that was refractory to massive fluid resuscitation and catecholamines [7-9,20]. Thus, vasopressin may be a simple and effective treatment for trauma patients who do not respond to advanced trauma life support, which may maintain arterial blood pressure at a level sufficient to ensure vital organ perfusion [7,8,20-22].

The data reported thus far about the type of fluid resuscitation [23-25], the target mean arterial blood pressure [23] and timing of fluid resuscitation timing [26] during advanced trauma life support are inconclusive [1]. However, it can easily be forgotten that certain patient groups, for example head trauma patients, require a certain level of cerebral perfusion pressure to prevent harm [27-29].

In our model, fluid resuscitation initially improved mean arterial blood pressure rapidly, followed by cardiovascular collapse and death in six out of seven pigs, owing to increased blood loss. This observation is in full agreement with findings in a porcine model of severe liver trauma resulting in uncontrolled haemorrhagic shock [5]. The underlying reason for excessive haemorrhage may be a dilution of blood clot factors, and dislodgement of newly created blood clots from the bleeding site [30]. In contrast, the underlying mechanism of terminated haemorrhage after vasopressin may be a potent vasoconstrictory effect via V_1 _receptors and therefore reduced blood flow in skin, muscle, fat tissue and gut. Accordingly, vasopressin shifts blood away from the bleeding side toward the heart and brain [21], thus decreasing bleeding and increasing vital organ blood flow.

Our observations in the fluid resuscitation group may suggest both advantages and disadvantages of our resuscitation protocols. Although an increasing end-tidal carbon dioxide level (+100%) in fluid-resuscitated animals indicated a significant increase in cardiac output, this increase in perfusion simply increased blood loss at the injury site, resulting in fatal blood loss. Haemodynamic development in our only surviving fluid-resuscitated animal lagged behind that in the nonsurviving fluid-resuscitated animals, which might have ensured survival. Accordingly, although fluid resuscitation is beneficial during controlled haemorrhagic shock, fluid resuscitation given during uncontrolled haemorrhagic shock may simply increase mean arterial blood pressure to a level at which the cardiovascular system becomes similar to an overflowing bath tub, reflecting ineffective therapy.

There are several limitations of our study that should be noted. Because this experiment had to be performed during general anaesthesia because of ethical considerations, the effects of anaesthetic agents such as propofol and piritramid might have had a confounding effect. We cannot state whether hypoperfusion of the gut results in organ damage, therefore limiting long-term survival. Also, we have no information about regional blood flow during experimental therapy, histological samples, or neurological outcome. Although a pulmonary artery catheter was placed inn the animals, we did not obtain cardiac output measurements because it was not possible to perform both multiple blood gas and cardiac output measurements simultaneously in this very dynamic model. We were unable to perform coagulation monitoring, and no fresh frozen plasma, thrombocytes, or clotting factors were administered because of limitations in laboratory and haematology resources. Furthermore, we did not employ blood transfusion, because this is not available in our emergency service. Also, we did not study the effect of a combination of vasopressin and fluid resuscitation in this study. Different vasopressin receptors in pigs (lysine vasopressin) and humans (arginine vasopressin) may result in a different haemodynamic responses to exogenously administered arginine vasopressin. Also, the present model reflects severe but local trauma; whether our experience can be extrapolated to patients with additional trauma, such as multiple fractures, requires investigation. Moreover, our pigs were intubated and undergoing continuous positive pressure ventilation throughout the experiment. Cardiopulmonary resuscitation was omitted in this model to allow us to compare fluid resuscitation with vasopressin treatment.

## Conclusion

Vasopressin but not fluid resuscitation or saline placebo ensured short-term survival in this vascular injury model of uncontrolled haemorrhagic shock in sedated pigs.

## Key messages

• Vasopressin treatment, but not fluid resuscitation or saline placebo, ensured short-term survival in this vascular injury model of uncontrolled haemorrhagic shock in pigs.

## Competing interests

In 2002 VW received a grant from Aguettant Laboratories (Lyon, France), a company that has applied for registration of vasopressin with the European authorities. There is no personal conflict of interest.

Data from a previous study [13] are being used for a vasopressin registration application process by Aguettant (Lyon, France) in Europe. Aguettant has supported our working group once with grant support in 2002. No author has a financial interest in drugs being discussed in this report.

## Authors' contributions

KHS designed the study protocol, conducted the laboratory work and wrote the manuscript. HGW helped to design the study protocol and was involved in laboratory work. ACK was involved in laboratory work and writing of the manuscript. WGV helped to design the study protocol and was involved writing of the manuscript. AK conducted all surgical work and was involved in the study design. KHL designed the study protocol and helped to interpret the data. VW designed the study protocol, supervised the laboratory work and was involved in writing the manuscript. All authors read the final draft of the manuscript and agreed with its content and data interpretation.
